# Acute Treatment of Osteochondral Detachment Following Patellar Dislocation: Clinical and Short-Term MRI Follow-Up

**DOI:** 10.3390/life14010085

**Published:** 2024-01-04

**Authors:** Leonardo Puddu, Giovanni Lugani, Francesco Perusi, Damiano Brunialti, Fabrizio Cont, Corrado Ciatti, Eleonora Poleggi, Leonardo Locatelli, Francesco Pisanu, Carlo Doria, Fabrizio Cortese, Gianfilippo Caggiari

**Affiliations:** 1 Orthopaedic and Traumatology Department, Santa Maria del Carmine Hospital, 38068 Rovereto, Italy; leonardo.puddu@apss.tn.it (L.P.); giovannilugani@gmail.com (G.L.); francesco.perusi@apss.tn.it (F.P.); fabrizio.cont@apss.tn.it (F.C.); fabrizio.cortese@apss.tn.it (F.C.); 2 Orthopaedic and Traumatology Department, University of Verona, 37126 Verona, Italy; damiano.brunialti@gmail.com; 3 Department of Orthopaedics, University of Sassari, 07100 Sassari, Italy; e.poleggi@studenti.uniss.it (E.P.); l.locatelli@studenti.uniss.it (L.L.); pisanuf@gmail.com (F.P.); cdoria@uniss.it (C.D.); gianfilippocaggiari@gmail.com (G.C.)

**Keywords:** patellar dislocation, osteochondral lesion, cartilage treatment, MRI, sports medicine

## Abstract

Background: The aim of our study is to emphasizes the significance of prompt diagnosis and intervention in younger patients affected by osteochondral detachment after patellar dislocation, where the first objective is to minimize in the shortest possible time complications and ingravescence. The method involves a clinical patient assessment and MRI follow-up in subjects who underwent to an immediate surgical intervention for osteochondral damage. Methods: From January 2020 to December 2022, 22 patellar dislocation cases were assessed; osteochondral lesions were identified in 12 (54%) patients; nine of these patients were treated immediately with knee arthroscopy, while in seven instances the osteochondral fragment was reattached using bioabsorbable pins. Post-operative clinical evaluations were conducted at one-, three-, and six-month intervals; finally, a six-month post-operative MRI was performed for all surgically treated patients. Results: The MRI evaluations, conducted six months post-operation for all seven patients, indicated successful integration of the reattached osteochondral fragment. Every patient returned to their pre-injury activities after surgery. However, two of them reported mild pain in the anterior region of the knee post-surgery. Conclusions: in young patients, swift diagnosis and immediate surgical intervention for osteochondral detachment resulting from patellar dislocation are crucial. This approach has been identified as the best practice, since it substantially minimizes immediate functional restrictions and significantly lowers the long-term risk of femoral-patellar osteoarthritis.

## 1. Introduction

Acute patellar dislocations frequently result from trauma, typically from non-contact twisting incidents involving the knee or direct impacts affecting the medial side of the knee. Habitual dislocations, on the other hand, represent as consistent dislocations every time the knee bends, where the main cause is represented by the excessive tightness of the vastus lateralis muscle and the iliotibial band [1]. Acute patellar dislocations emerge as a common injury during early adolescence, with an incidence rate spanning from 29 to 42 out of every 100,000 children below the age of 16 [2,3]. These injuries have predominantly traumatic origins, caused either by direct blows to the knee or twisting motions [4].

Certain anatomical variations, such as patella alta, trochlear dysplasia, and an elevated TT-TG distance, which gauges the gap between the tibial tubercle and the trochlear groove [4], can heighten the risk of patellar dislocations. Ligamentous laxity, more commonly observed in females or those with underlying connective tissue disorders, also increases the chances of dislocation. A muscular imbalance, especially a weakened vastus medialis oblique, contributes notably to patellar instability. In patients without such predispositions, acute dislocation episodes are more likely associated with osteochondral lesions. This is due to a more forceful trauma needed to initiate the dislocation, producing significant shear forces on the patella’s cartilaginous surface.

Our study aims to underscore the importance, in all young patients diagnosed with patellar dislocation, of an early diagnosis and subsequent acute surgical intervention. These measures can reduce the complications that may occur with diagnostic and therapeutic delay, ensuring immediate recovery of joint function and preserving it over time. Patellar instability ranks as the predominant knee condition among children and adolescents. Undergoing surgical intervention substantially lowers the chances of recurrence, yet the inherent conditions and the nature of the instability can influence the outcomes [5]. Despite the difficulty in classifying patellar instability in children and adolescents due to the comorbidity of associated pathologies and skeletal immaturity, currently, one can refer to the classification of Parikh and Lykissas, which divides the patellar instability into four types, in order of increasing severity (Table 1).

Analyzing the epidemiology of the different types of patellar instability in Table 1, it is important to underline how each dislocation is more found in subjects of different ages than in older adults. Type 1 and type 2 dislocations are found more frequently among adolescents, type 3 is mostly congenital, while type 4 dislocations are less frequent [6].

After episodes of patellar dislocation, patellar osteochondral lesions, as initially documented by Kroner in 1905 [7], are more frequently detected via magnetic resonance imaging (MRI). Detachments of osteochondral matter typically manifest at the patella’s inferomedial pole and the lateral femoral condyle’s outer edge. However, these two lesions are frequently concurrent.

In the early days of 1977, Cofield and his colleagues reported a 14% occurrence rate of osteochondral lesions from a study analyzing 50 patients with acute patellar dislocations. For younger patients, aged between 13 and 18, the incidence rate of such lesions can reach up to 71%. This is especially true for those with inherent ligamentous laxity, often facing more frequent and recurrent patellar dislocations than other patients [8].

The trauma mechanism leading to osteochondral detachment following an acute patellar dislocation, stems from the shear forces exerted on the patella’s medial side and the lateral femoral condyle. For a thorough diagnosis of patellar osteochondral lesions, beyond objective examinations and clinical evaluations, first-level instrumental exams are paramount. All affected individuals should undergo X-ray examination. Considering the case of X ray positivity to possible fractures or osteochondral detachments, CT scans provide further diagnostic clarity and should be performed [9].

However, solely relying on standard X-rays can lead to overlooked osteochondral lesions, even though it is advisable to use MRI, especially in patients with innate ligamentous laxity. In these cases, osteochondral lesions are rarer due to reduced shear forces on cartilage surfaces. The treatment strategy varies based on lesion size, type, fragment count, and mobility, and details were obtained via arthroscopy. This procedure not only assesses the extent of osteochondral damage, but also the fragment’s characteristics, guiding surgeons on whether reinsertion is feasible or if removal and subsequent regenerative techniques are needed for a better and more stable healing of the fragment.

In general, if a fragment retains its structure and size suitable for reinsertion, this method is favored to reduce early osteoarthritis risks. Only in scenarios where successful in-joint stabilization is improbable to obtain the fragment should be considered for removal [10,11].

We believe that this study could be important and useful for orthopedic specialists, as this topic is still little debated in the current literature, despite being a pathology to take into consideration mostly in younger patients.

## 2. Materials and Methods

Between January 2020 and December 2022, 22 patients, being 8 males and 14 females, aged between 12 and 17 years, were admitted to the Emergency Department of Ospedale Santa Maria del Carmine in Rovereto with a diagnosis of acute patellar dislocation.

**Figure 1:**
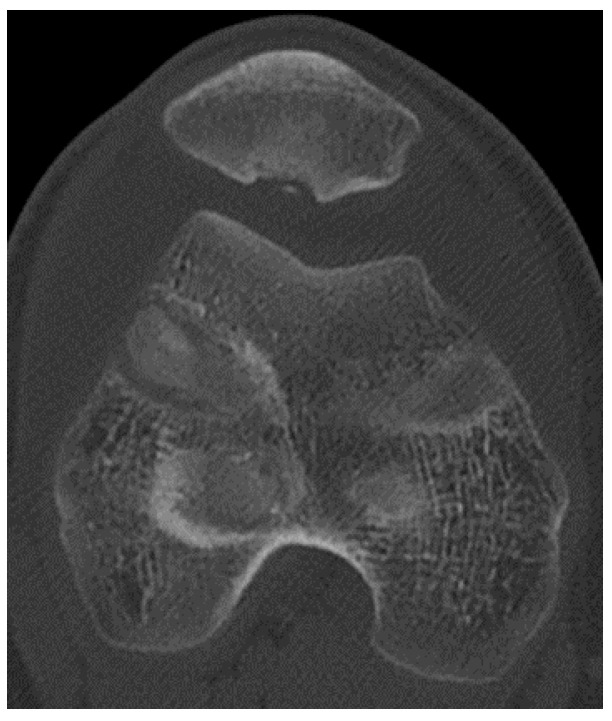
CT scan image of an osteochondral lesion of the patella.

In the Emergency Department, all patients underwent first-level instrumental examinations: a first line X-ray examination was performed, and those patients with a suspected fracture or osteochondral detachment on the X-ray imaging were further on evaluated with CT scan diagnostics (Figure 1).

All 22 patients were acutely treated with reduction and immobilization of the dislocation in a femoral–malleolar brace. After that, they underwent magnetic resonance (MRI) and an orthopaedical evaluation as follow-up was scheduled.

Our data shows that in up to 12 out of 22 total patients (54.5%), the MRI exam showed the presence of an osteochondral lesion, with or without the presence of an intra-articular fragment related to the mobilization of the native lesion.

**Figure 2:**
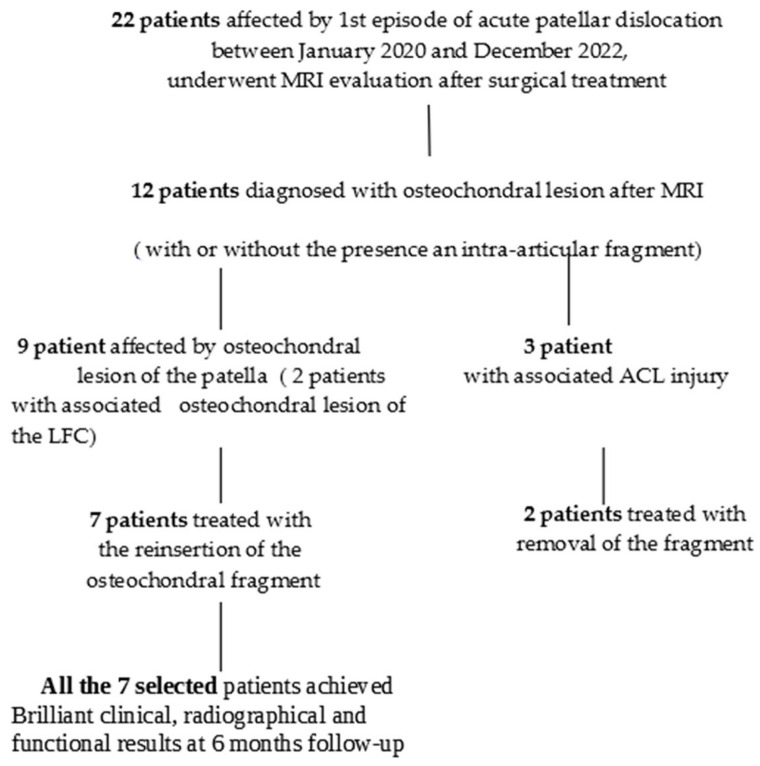
Flow chart of the patients selected for the retrospective observational study.

Twelve patients with patellar osteochondral lesions underwent knee arthroscopy within 3 weeks after the trauma to manage the osteochondral detachment and any associated injuries. Furthermore, among the 12 patients with an osteochondral lesion, 3 patients (25%) had an associated anterior cruciate ligament (ACL) injury and 2 of them (17%) presented an osteochondral lesion of the lateral femoral condyle. The 3 patients with associated ACL injuries were excluded from the retrospective study. Considering the group of 9 selected patients consisting of 3 males and 6 females, aged between 14 and 17, two patients reported an Osteochondral lesion of the lateral femoral condyle (LFC). Between the 22 patients, in 7 of them, an osteochondral lesion greater than 1.5 cm^2^ was observed on magnetic resonance imaging, and two patients presented a lesion with dimensions less than 1.5 cm, where the osteochondral fragment was not reinserted due to its size, fragmentation, and position. In the remaining 7 patients, the reinsertion of the osteochondral fragment was possible using absorbable pins, thanks to this condition the remaining 7 patients formed the retrospective observation group of this study (Figure 2) with demographic characteristics listed in Table 2.

## 3. Diagnostic Technique

### 3.1. Plain Radiographs (X-rays)

For an accurate clinical assessment of patients presenting with knee-related issues, initiating the diagnostic journey with a standard X-ray is essential. Procuring both anteroposterior (AP) and lateral radiographs of the afflicted knee is fundamental, supplemented by detailed patellar views. These images play a pivotal role in spotting fractures, pinpointing loose bodies, identifying malalignment, or highlighting any arthritic manifestations of any kind. Typically, fractures or floating bodies emanate from the patella’s medial side, occasionally extending to involve the lateral femoral condyle. The lateral radiographic view offers an insightful analysis of trochlear dysplasia. The presence of the ‘crossing sign’ when the trochlear groove aligns with the anterior boundary of the lateral condyle indicates possible flattening. Moreover, the ‘double contour sign’ emerges when there is a convex trochlear groove or an underdeveloped medial condyle.

### 3.2. Computer Tomography (CT)

Scans throughout our in-depth study, instances were noted where patients’ X-rays exhibited ambiguous images, suggestive of fractures or osteochondral detachments. In such scenarios, a CT scan became imperative to enrich the diagnostic clarity to better understand the overall picture. CT scans provide an efficient means to measure the TT-TG distance, an integral metric when evaluating and managing patellar dislocations. To discern this, a tangent line is drawn connecting the posterior borders of both femoral condyles. Subsequently, two perpendicular lines are marked: one (A) from the tibial tubercle’s apex, and the other (B) from the trochlear groove’s deepest point. The spacing between lines A and B is the TT-TG distance. A standard measurement stays below 20 mm, with anything surpassing this limit deemed anomalous. Beyond this, CT scans adeptly outline the presence, depth, and extent of osteochondral fractures, equipping medical professionals with insights to strategize potential most suitable surgical interventions [12,13].

### 3.3. Magnetic Resonance Imaging (MRI)

MRI emerges as an invaluable tool in evaluating the intricate soft tissue structures encompassing the knee. In situations involving complete dislocation, the diagnostic image often reveals a distinct bruising pattern localized to the lateral femoral condyle and the medial section of the patella. This advanced imaging technique excels at spotting articular cartilage damage, especially on the medial patellar facet, and boasts superior sensitivity in detecting osteochondral lesions compared to the other methods previously mentioned. Furthermore, MRI offers a comprehensive view of the trochlear anatomy, especially in identifying trochlear dysplasia. As our study’s findings underline, MRI is indispensable in detecting patellar osteochondritis lesions that might develop post patellar dislocation episodes. This diagnostic tool proves to be particularly beneficial for patients with inherent ligamentous laxity. Within the confines of our research, MR imaging stood out as a linchpin, ensuring precise diagnosis and efficient post-surgical patient follow-up.

### 3.4. Surgical Technique

The arena of surgical intervention, especially when addressing osteochondral lesions, does not abide by a monolithic treatment strategy. Several considerations play pivotal roles in determining the optimal treatment pathway, including predisposing factors for patellar dislocation and the intrinsic details of the osteochondral fragment, which are predominantly ascertained through the lens of arthroscopy.

In the all-important preoperative evaluation phase, understanding the trauma’s genesis is merely the tip of the iceberg. The surgeon must also deeply assess the patient’s functional requirements and discern the presence or absence of predisposing elements that could escalate the risks of recurrent patellar dislocations, which are synonymous with patellar instability and therefore a faster degeneration to an osteoarthritic context. The osteochondral fragment, with its diverse size range, positional nuances, and unique characteristics, requires an initial, thorough arthroscopic evaluation. Such assessment is paramount in guiding the surgeon’s decision, whether leaning towards reinserting the fragment or advocating its removal, especially in situations where the former might be unfeasible.

Diving deeper into the surgical procedure’s intricacies, post the completion of anesthesia induction, the patient assumes a supine position on the operating table. A precursor to the primary surgical maneuver involves assessing and gauging the degree of patellar hyperlaxity, a direct consequence of any injury to the medial patellofemoral ligament (MPFL). Arthroscopy is invariably the first step in the surgical playbook, primarily due to its unmatched efficacy in determining the osteochondral damage’s topography and magnitude. It provides the surgeon with a vivid, real-time visual vantage point, enabling them to make judicious intraoperative decisions on the fragment’s disposition and size, keystone for deciding whether a reinsertion or a removal conundrum is needed.

**Figure 3:**
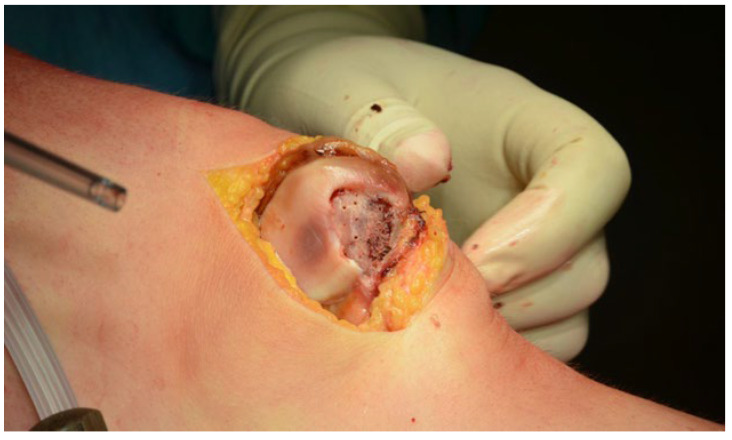
Preparation of the subchondral bone through microperforations.

**Figure 4:**
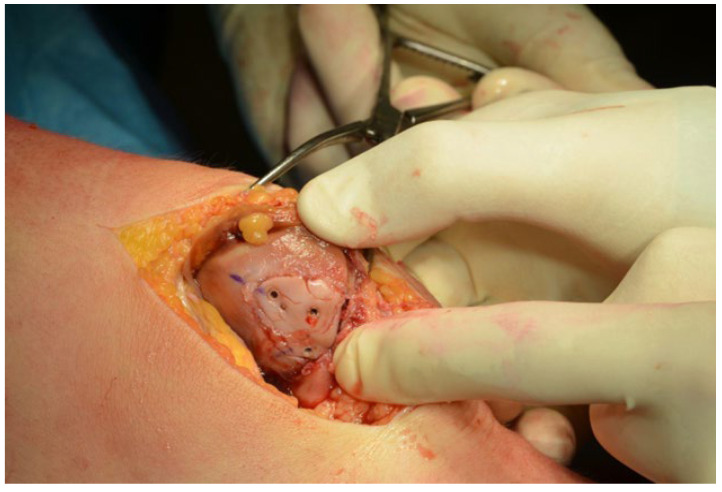
Reinsertion of the osteochondral fragment using absorbable pins.

A significant facet of the arthroscopic procedure entails the potential pinpointing or validation (should prior MRI evidence be available) of concomitant chondral lesions, notably on the lateral femoral condyle. Detected lesions can undergo arthroscopic management, most commonly leveraging the microfracture technique [12]. Drawing from the extensive data collated in our study, we discerned a pattern. Patients, where arthroscopic revelations manifested osteochondral fragments with a surface area exceeding 1.5 cm^2^, typically steered towards reinsertion of the fragment into its indigenous site, which helped elaborating further on the following surgical paradigm:

1. Medial arthrotomy for enhanced exposure: The inaugural step involves a strategic medial arthrotomy. This methodological incision provides surgeons with unparalleled access to the patellar chondral surface. Furthermore, it also facilitates the repair of the compromised medial patellofemoral ligament, which is a critical structure to ensure patellar stability.
2. Chondral fragment refinement: Upon extraction, the chondral fragment often necessitates adjustments. Trauma may alter its size or morphology, making it imperative to reshape the fragment. This meticulous reshaping ensures the fragment’s snug fit when reintroduced to its original lesion area to provide a more accurate reduction and better healing.
3. Cartilage surface priming and microperforations: Before the reinsertion of the fragment, the existing cartilage surface undergoes rigorous preparation. This encompasses the thorough debridement of the subchondral bone. If clinically advised, microperforations are executed to optimize the lesion site’s regenerative potential. Visual references for this stage are often illustrated in Figure 3.
4. Fragment reinsertion and fixation: With the lesion area primed and the fragment suitably reshaped, the next logical step is its reinsertion in the original site. This process is not merely a reintroduction, but involves securing the fragment to ensure its stability. Absorbable pins, exemplified in Figure 4, have gained favor for this purpose due to their biocompatible nature.
5. Medial Patellofemoral Ligament (MPFL) intervention and patellar tracking: There is a subset of patients whose preoperative imaging (MRI or CT) indicates significant patellar tilt, often exceeding 12°. For such individuals, the repair of the Medial Patellofemoral Ligament becomes non-negotiable. Additionally, to further enhance patellar tracking and reduce the risk of future dislocations, a medial plastic procedure, benchmarked against the renowned Insall technique, is employed.

Post-surgery, the recovery roadmap is equally structured. Adhering to a regimented postoperative protocol is imperative to ensure a return to pre-injury activities. The immediate aftermath of the surgery sees the patient’s knee immobilized using a specialized brace. This immobilization is coupled with touch weight-bearing directives for the surgically treated leg, allowing for a restrained knee motion range between 0–30°. This conservative approach spans two weeks. After this phase, there is a gradual increase in the knee’s flexion, marked by weekly increments of 30°. This progression continues for a month post-surgery, ensuring optimal healing and functional restoration.

## 4. Results

During a comprehensive research study, our team investigated the clinical outcomes of patients subjected to arthroscopy due to osteochondral lesions following patellar dislocation. A cohort of nine patients was examined, with two undergoing fragment removal and the remaining seven benefiting from reinsertion of the osteochondral fragment.

The patients, especially those with reinserted fragments, underwent regular clinical monitoring at 1, 3, and 6 months postoperatively. A key aspect of the 6-month follow-up was an MRI scan. One patient presented a unique challenge, having a significant grade 3 IKDC lesion of 1.5 cm² in the lateral femoral condyle’s weight-bearing area, which was treated with the Steadman microfracture technique [14].

The literature also describes other surgical techniques apart from ours. Repo et al. describe in their article a similar scenario where their surgical technique considers the fixation of the lesion fragment with biodegradable pins only if its size is larger than 1 cm² and located on the weight-bearing surface of the patella, otherwise the fragment is removed arthroscopically. They also utilize microfractures and decided to reconstruct the MPFL with grafts from adductor magnus, gracilis, or semitendinosus tendons [15]. Scanlon et al. analyzed their approach to a pool of 19 patients affected by patellar dislocation associated with osteochondral defect all being treated with MPFL reconstruction. Their surgical technique consists of a first arthroscopic examination of the fragment via standard anterior portals to better understand the general picture, and, in the case of chondral fragments amenable to fixation, a medial parapatellar approach led to the patellar lesion and a lateral arthrotomy led to condyle and femoral defect to be repaired. A superficial quadriceps tendon graft was then harvested and pulled throughout a tunnel drilled in the retinaculum and the patella, to then secure the patella with the femur [16].

Postoperative progress assessments focused on functional recovery, using the Kujala Anterior Knee Pain Scale score, a 13-question self-assessment tool measuring subjective experiences of activities and symptoms associated with anterior knee pain. Interpretative metrics range from 0 to 100, with 100 signifying no pain and optimal knee function.

**Figure 5:**
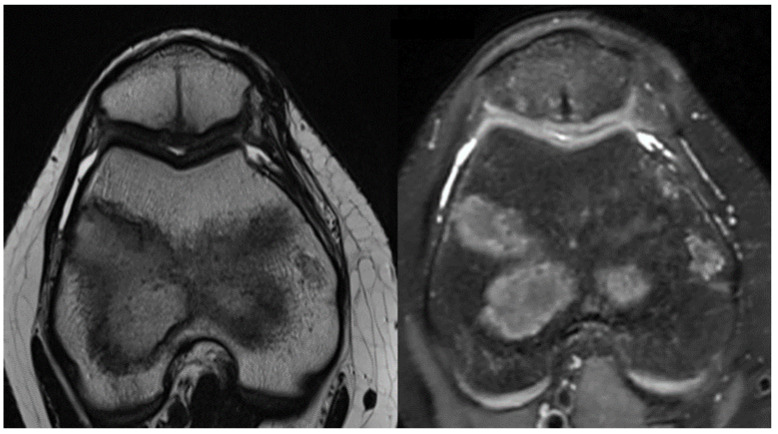
Six months follow-up MRI images that show complete filling of the osteochondral lesion of the patella.

Six months after surgery, MRI scans were evaluated using the MOCART Score to categorize healing progress of the osteochondral defects (Figure 5). Recovery classifications ranged from subchondral bone exposure to full lesion coverage, indicating complete healing. The outcomes at the 6-month interval were positive, with Kujala Scale scores reaching above 90 points and MRI evaluations showing no reinsertion failures. Impressively, complete lesion filling was observed in two cases, highlighting the success of the surgical procedures [17,18].

Initially, all 22 patients were treated with dislocation reduction and immobilization. Subsequent MRIs revealed osteochondral lesions in 54.5% of patients, with some also presenting intra-articular fragments. These patients underwent arthroscopy within three weeks of the trauma. Among them, those with additional ACL injuries were excluded from this retrospective study.

The selected nine patients, aged between 14 and 17, included three males and six females. Seven of these exhibited osteochondral lesions larger than 1.5 cm² on MRI. In two patients out of the ones with smaller lesions, the fragments were not reinserted due to size, fragmentation, and position. However, in the other seven patients, reinsertion was successful using absorbable pins. These individuals constituted the retrospective observation group of the study, and their demographic characteristics are detailed in Table 3.

## 5. Discussion

Echoing the findings from Nomura et al., a staggering 95% of patients suffering from an acute patellar dislocation event encounter cartilage impairment. The patella’s inferomedial pole is often the most jeopardized, with the lateral femoral condyle getting affected in about 31% of cases. Moreover, simultaneous lesions in both regions are not uncommon [19,20].

The consequences of osteochondral damages post-patellar dislocation are multifaceted. In the immediate aftermath, patients face severe functional impediments. The longer-term ramifications are even more concerning, with a dramatic escalation in femoral-patellar osteoarthritis risks. Hence, swift diagnosis and a tailored intervention strategy are pivotal for an optimal patient outcome and healing.

Our study focuses on the importance of second-level instrumental diagnostics in young patients with patellar dislocation and its timely use, followed by surgery.

This is demonstrated by the extremely positive results obtained by us; in fact, in our sample of interest it emerged that among the 22 patients examined with acute patellar dislocation, 12 showed through the timely execution of MRI an osteochondral lesion of the patella, subsequently they were treated surgically in a timely manner; specifically, in seven patients surgery that led to the reinsertion of the fragment was performed. In the follow up, all seven patients showed outstanding radiographic and functional clinical results. This has been possible through the use in a favorable time window of the MRI, with the aim of obtaining in a short time a framework on the clinical condition of the patient, and with it the possibility of performing an acute surgical treatment, thus drastically reducing medium and long-term complications and recovery of immediate functionality.

CT or MRI findings revealing chondral or osteochondral lesions, particularly in scenarios with floating joint fragments, they accentuate the necessity of surgical intervention, especially in younger patients with high functional demands such as active and sport youngsters. A plethora of research underscores the superior clinical returns of osteochondral fragment reinsertion over long durations, provided its feasibility [21].

To orchestrate the best surgical strategy, a meticulous pre-operative evaluation is imperative. Here, beyond the trauma dynamics, it is crucial to gauge the patient’s functional aspirations and identify any inherent triggers for recurring patellar dislocations, thereby contributing to patellar instability. Key markers like patellar height, trochlear dysplasia, and TT-TG index evaluations can provide valuable insights [22].

A singular acute patellar dislocation episode can instigate osteochondral lesions, especially in the absence of inherent patellar instability risk factors. Such incidents usually arise due to intense trauma, inflicting amplified shear stress on the patella’s cartilage layer. This reinforces the mandate for an advanced diagnostic procedure like MRI. As a protocol, MRI should be the primary investigative approach for all patellar dislocation cases. However, if initial X-rays after a patellar dislocation incident yield ambiguous findings regarding osteochondral impairments, an emergency CT becomes indispensable [23].

The osteochondral fragment’s characteristics, discerned through preliminary arthroscopic evaluations, guide the treatment choice between reinsertion or removal of the fragment. The literature cements the superiority of fragment reintegration over other interventions for medium to long-term results. Gesslein et al.’s retrospective analysis, contrasting patients treated with osteochondral fragment reinsertion against those undergoing debridement or microfractures, found the former group to exhibit notably superior clinical metrics during extended follow-ups [24].

From a surgical standpoint, a one-size-fits-all approach is elusive. Arthroscopy remains the minimally invasive gold standard, beneficial for both osteochondral injury detection and lateral femoral condyle treatments. In scenarios where fragment reinsertion is not obligatory, surgeons often lean on regenerative arthroscopic techniques, like microfractures. For cases mandating osteochondral fragment reinsertion of the lateral femoral condyle, arthroscopic measures are sufficient. Regardless of the fragment’s inherent location, absorbable fixation devices emerge as the preferred tool for its reinsertion [25,26].

However, scenarios demanding a parapatellar approach necessitate a more intricate, open surgical route via medial arthrotomy. This strategy caters to both the patellar fragment reinsertion and concurrent repairs of the medial patellofemoral ligament, which accentuates MRI’s indispensability, not only as first diagnostics, but also during follow-ups. This imaging modality offers unparalleled insights into treatment efficacy, reinserted fragment stability, and defect filling extents. For patients treated with fragment reinsertion, a 6-month post-surgery MRI is recommended unless clinical observations suggest fragment dislodgements [27].

While multiple clinical scores offer a lens into cartilage quality, it is paramount to remember that such scores might not always mirror the actual cartilage state, underscoring the need for holistic, multi-modal evaluations [28].

## 6. Conclusions

Following an incident of patellar dislocation, magnetic resonance imaging (MRI) emerges as a critical diagnostic tool. Its significance is anchored in its unmatched ability to pinpoint osteochondral lesions that might demand immediate surgical intervention. The comprehensive imaging provided by MRI ensures that healthcare practitioners can grasp the full scope of the injury, allowing them to devise the most effective treatment plan for each patient.

Prompt surgical intervention for osteochondral detachment after a patellar dislocation is widely recognized as the optimal course of action, particularly for younger individuals with high functional demands. This approach transcends just treating the evident damage. It is a strategic move aimed at pre-empting future complications. By adopting this acute surgical treatment, the risks associated with short-term functional challenges can be significantly mitigated. Even more importantly, this proactive measure plays an essential role in dramatically reducing the likelihood of patients developing femoral-patellar osteoarthritis as they age.

Despite the favorable data collected, our study presents several limitations due to the small size of the sample under examination, the absence of a control group and the short post-operative follow-up. Also, an important limitation was the failure to analyze Kujala’s scores on patients prior to surgery.

In conclusion, while MRI provides an in-depth and precise diagnostic insight post-patellar dislocation, acute surgical solutions offer a path of timely intervention. For younger patients who lead active lives, this combination of accurate diagnosis and swift surgical action ensures the most favorable outcomes, both in terms of immediate recovery and in preserving long-term knee functionality.

## Figures and Tables

**Table 1:** Classification of patellar instability according to Parikh and Lykissas.

Type	Subtype
Type 1 First patellar dislocation	A With osteochondral fracture
	B Without osteochondral fracture
Type 2 Recurrent patellar instability	A Recurrent patellar subluxation
	B Recurrent (>2) patellar dislocation
Type 3 Dislocatable patella	A Passive patellar dislocation
	B Habitual patellar dislocation in flexion or extension
Type 4 Dislocated patella	A Reducible
	B Irreducible

**Table 2:** Demographic and anatomopathological characteristics of the patients.

	Age	Sex	Associate Lesions	Osteochondral Lesion Size (cm^2^)	Treatment
**1**	14	M	NO	>1.5	Reinsertion
**2**	15	F	NO	>1.5	Reinsertion
**3**	17	F	NO	>1.5	Reinsertion
**4**	16	F	YES *	>1.5	Reinsertion
**5**	17	M	NO	>1.5	Reinsertion
**6**	17	F	NO	>1.5	Reinsertion
**7**	16	M	NO	>1.5	Reinsertion
**8**	17	F	YES *	<1.5	Asportation
**9**	17	F	NO	<1.5	Asportation

* Osteochondral lesion of the lateral femoral condyle (LFC).

**Table 3:** Clinical and MRI results at 6 months follow-up.

	Age	Sex	Kujala Scale	Fill of the Lesion MRI
**1**	14	M	94	>50%
**2**	15	F	98	>50%
**3**	17	F	100	100%
**4**	16	F	92	<50%
**5**	17	M	90	<50%
**6**	17	F	96	100%
**7**	16	M	94	100%

## Data Availability

The original contributions presented in the study are included in the article, further inquiries can be directed to the corresponding author.
